# Non-surgical treatment of giant tumor-like lumbar disc herniation based on enhanced MRI: A case series

**DOI:** 10.1097/MD.0000000000032594

**Published:** 2023-01-13

**Authors:** Xueqiang Shen, Shun Lin, Hong Jiang, Jintao Liu, Pengfei Yu

**Affiliations:** a Suzhou TCM Hospital Affiliated to Nanjing University of Chinese Medicine, Suzhou, Jiangsu Province, China; b Nanjing University of Chinese Medicine, Nanjing, Jiangsu Province, China.

**Keywords:** enhanced MRI, lumbar disc herniation, resorption, tumor-like

## Abstract

In recent clinical follow-up, it has been vertified that resorption in lumbar disc herniation (LDH) could be of great curative effect in non-surgical treatment for LDH. However, reports of resorption in giant tumor-like LDH are rarely mentioned due to its risk of irreversible neurological damage which could be caused by long-term non-surgical treatment. In our clinical observations, we have found that enhanced MRI helps to distinguish LDH from intradural tumours and to predict the probability of resorption in LDH. We analyzed 8 patients with giant tumor-like LDH who underwent non-surgical treatment, and these patients had resorption during follow-up. All patients were examined with enhanced MRI before treatment, and the type of “bull’s eye” sign classification was determined by images. The MRI protrusion volume(VP), resorption rate(HR%) and JOA score of patients at the first visit and the last follow-up were recorded.

**Patient concerns:**

8 patients of Han ethnicity were admitted to the department of orthopedic complaining of low back pain for 1week to 12months. They were diagnosed with giant tumor-like LDH by enhanced MRI.

**Diagnoses:**

These patients were diagnosed with giant tumor-like LDH.

**Interventions:**

We adopted a non-surgical treatment plan for the patients, including taking oral non-steroidal anti-inflammatory agents and performing rehabilitation exercise. In consideration of the risk of irreversible neurological damage, patients were closely observed during treatment and follow-up. Once the following conditions occur, surgical treatment is required immediately: The symptoms are not signifcantly relieved after 3 to 6 months of non-surgical treatment; The symptoms are aggravated by non-surgica treatment; The clinical manifestations of cauda equina syndrome.

**Outcomes:**

After treated with oral non-steroidal anti-inflammatory agents and rehabilitation exercise, the resorption was accompanied by clinical symptom relief. No neurological damage occurred in all patients, and the clinical symptoms did not recur in the subsequent follow-up.

**Lessons:**

Clinicians should fully consider the possibility of resorption prior to surgical treatment in patients with giant LDH. We can predict the probability of resorption in patients with giant LDH based on enhanced MRI. For patients with a high probability of resorption, we can choose non-surgical treatment in the absence of progressive neurological impairment and cauda equina syndrome.

## 1. Introduction

Since Guinto et al ^[1]^ first identified the phenomenon of “disk regression” in herniated intervertebral disc through computed tomography follow-up, in 1984, scholars have conducted in-depth research on resorption of lumbar disc herniation (LDH) in the last 20 years, and the understanding of this phenomenon has made great progress. Without surgical intervention, the herniated disc shrinks or disappears, accompanied by significant improvement in clinical symptoms, which provides the most direct basis for the non-surgical treatment of LDH.^[2–4]^ However, there are few reports of resorption in giant tumor-like LDH.

It is relatively difficult to distinguish giant tumor-like LDH from intraspinal tumors (spinal cord lipoma, spinal chordoma, intraspinal schwannoma, malignant peripheral nerve sheath tumors and spinal neurofibroma) by non-surgical and noninvasive methods. Although magnetic resonance imaging (MRI) is considered as the most appropriate noninvasive test to confirm the presence of LDH, it is still of possibility to mix up intraspinal tumors with LDH by simple MRI. Enhanced MRI plays an important role in the clinical diagnosis and treatment of LDH: For one thing, it helps make a distinction with giant tumor-like LDH and intraspinal tumors,^[5]^ for another the “bull’s eye” sign (refers to the circumferential ring enhancement of the herniated disc tissues caused by the growth of new blood vessels) on enhanced MRI is valuable for the prediction of LDH resorption.^[6,7]^

## 2. Materials and methods

### 2.1. Study subjects

This is a retrospective observational study carried out at the Suzhou TCM Hospital Affiliated to Nanjing University of Chinese Medicine (Jiangsu, China) from March 2011 to June 2020. Eight patients with giant tumor-like LDH who had resorption after non-surgical treatment formed the basis of this study. The clinical data, including clinical symptoms, MRI images, Japanese orthopaedic association (JOA) score before and after treatment, “bull’s eye” sign classification, follow-up time, time of resorption and other clinical data were collected from case notes. According to the clinical symptoms and MRI imaging findings, these 8 patients were diagnosed as giant tumor-like LDH. We adopted a non-surgical treatment plan for the patients, including taking oral non-steroidal anti-inflammatory agents and performing rehabilitation exercise. In consideration of the risk of irreversible neurological damage which could be caused by long-term non-surgical treatment, patients are closely observed during treatment and follow-up. Once the following conditions occur, surgical treatment is required immediately ^[8]^: The symptoms are not signifcantly relieved after 3 to 6 months of non-surgical treatment; The symptoms are aggravated by non-surgica treatment; The clinical manifestations of cauda equina syndrome.

### 2.2. Measurement of MRI volume of protrusion (VP) and resorption rate (RR%)

**Figure 1.**
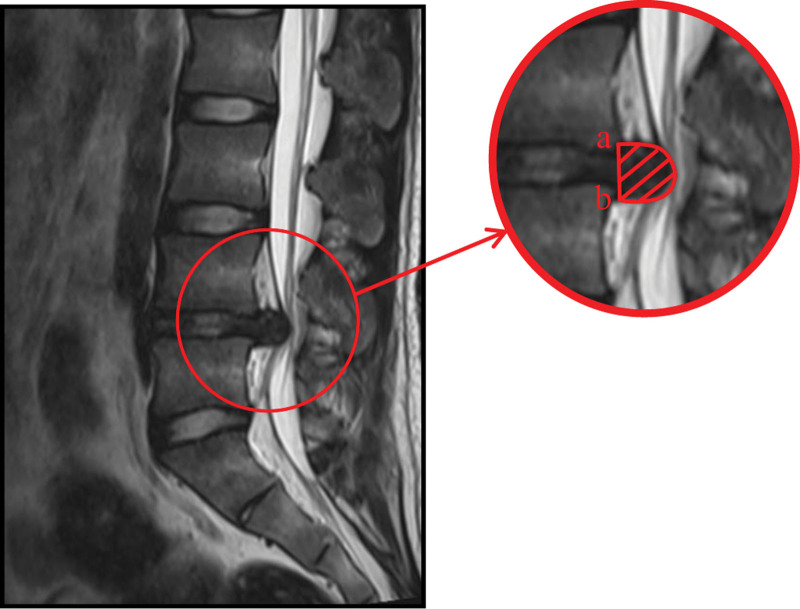
Schematic diagram for measurement of MRI protrusion area. On plain MRI, T2WI sagittal images, lower edge of the upper vertebral body (a), upper edge of the lower vertebral body (b), and section in red indicates the protrusion area. MRI = magnetic resonance imaging.


We measure the RR% by calculating the volume change of the herniated disc in MRI before and after treatment. MRI VP measurement was performed using a Siemens 1.5T superconducting MRI unit (magnetoionic intensity: 0.35 Tes/a; spin-echo sequence; 11 layers scanned at the sagittal position; interlayer spacing: 1.25 mm; layer thickness: 5 mm). The image data were processed using picture archiving and communication systems (PACS). We measure the volume of the protrusion on PACS software according to the method described by Dai.^[9]^ On the PACS software, we selected T2WI sagittal MRI images. The rear connection of the lower edge of the upper vertebral body and the upper edge of the lower vertebral body were used as the inner boundary; the rear edge of the herniated disc serves as the outer boundary; and the area of the herniated disc was then calculated using software (Fig. 1). We calculate the volume of herniated disc (VP, mm^3^) by the following formula^[9]^:

$$\begin{array}{l} VP=(IS+LT)\times \underset{i}{\overset{n}{\sum }}\;APi \end{array}$$

*IS* represents interlayer spacing, *LT* represents layer thickness, *APi* represents the area of the herniated disc of layer *i*, and the total layer number is *n*.

The RR% was calculated by the equation:


$$\begin{array}{l} RR\%=\frac{VP1-VP2}{VP1}\times 100\% \end{array}$$

*VP*1 represents volume of herniated disc brfore treatment, *VP*2 represents volume of herniated disc after treatment.

### 2.3. Measurement of herniated area as percentage of corresponding vertebral canal area (AR%)

**Figure 2.**
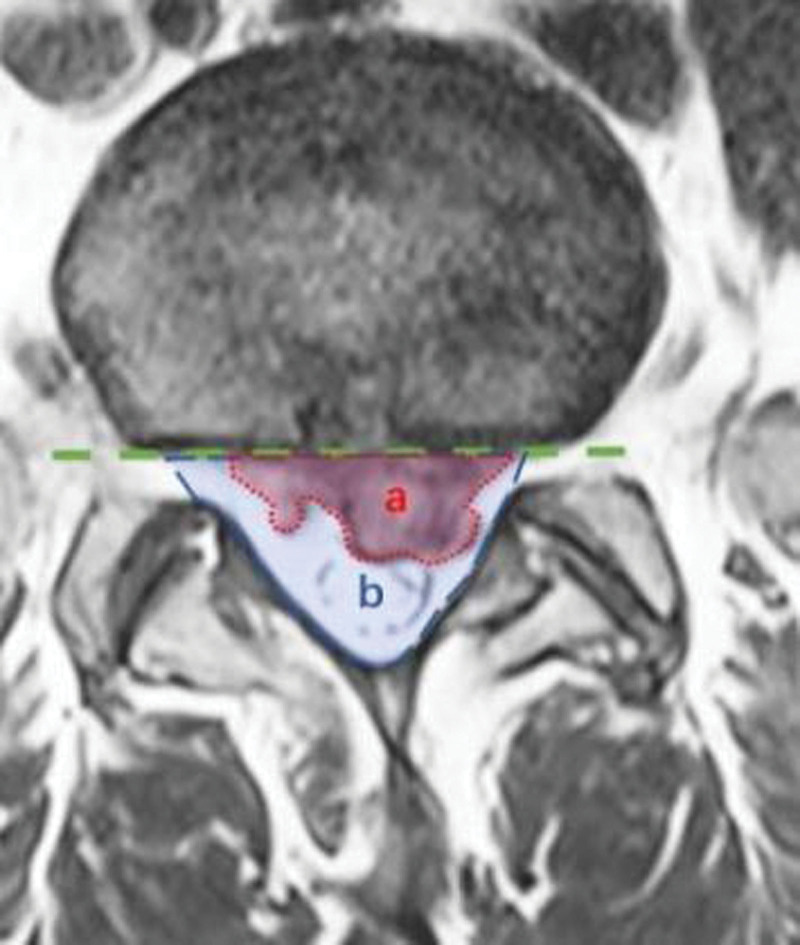
Schematic diagram for measurement of protrusion horizontal area. On plain MRI, T2WI horizontal images, datum line on the anterior wall of spinal canal(*l*), protrusion area (a), and spinal canal area (b). MRI = magnetic resonance imaging.

On the PACS software (Shenyang, Liaoning Province, China, Version5.5), we selected the T2WI horizontal MRI image with the largest area of the herniated disc. We make a datum line on the anterior wall of spinal canal and trace the herniated disc area **a**, spinal canal area **b** on the software (Fig. 2). The ratio of **a** to **b** is AR%.

### 2.4. Evaluation of clinical symptoms (JOA score) and statistical analysis

The improvement of clinical symptoms was evaluated by JOA score before and after treatment. Continuous variables were described using arithmetic means and standard deviations (SD). The JOA score, VP and AR% before and after treatment were performed by the paired *t*-test. All statistical analyses were performed by SPSS (version 24.0). *P*-value < .05 was considered statistically significant.

## 3. Results

### 3.1. Patients’ characteristics

Our cases included 5 men and 3 women, aged 20 to 51 years, with an average of (40.63 ± 10.20) years. The segments of disc herniation included L_4/5_ 6 cases and L_5_/S_1_ 2 cases. Among the patients, there were 6 cases of type A “bull’s eye” sign classification, 2 cases of type B, and no type C. Course of disease from 1 week to 1 year, with an average of (3.50 ± 4.25) months.

### 3.2. MRI imaging outcome

**Figure 3.**
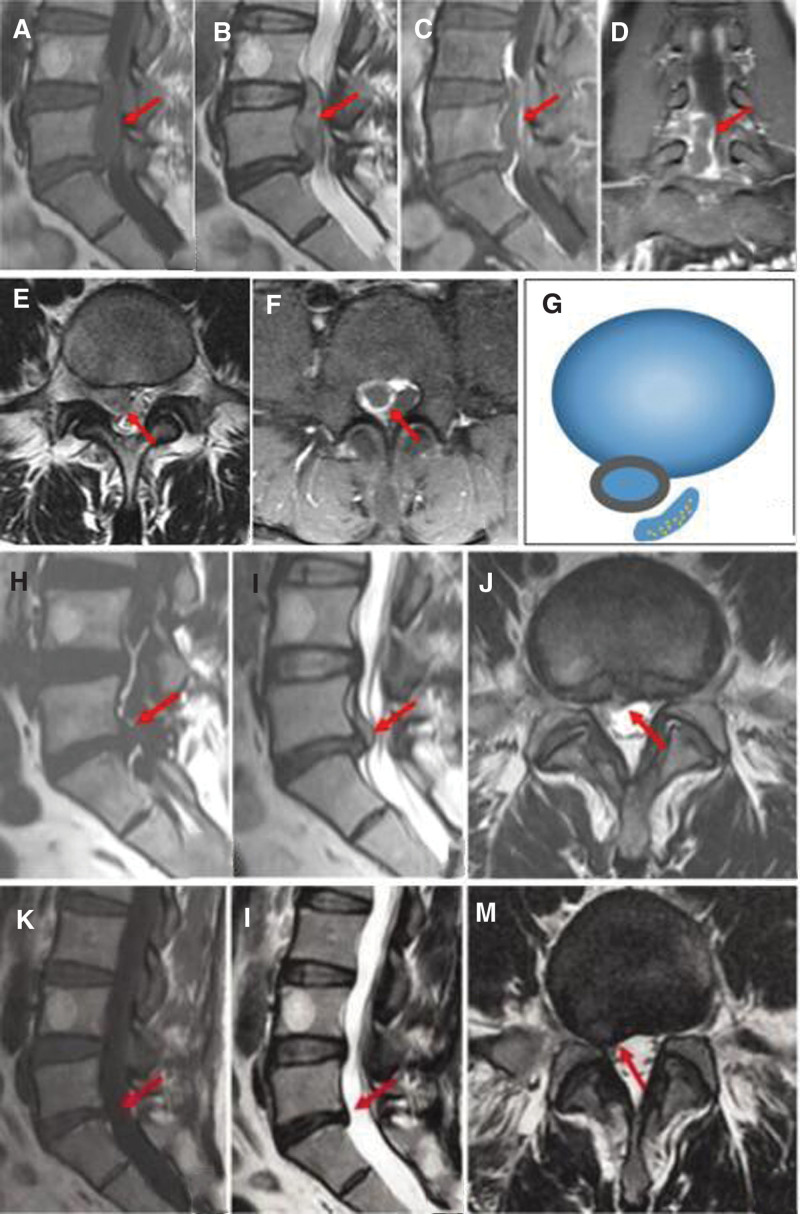
MRI images of a 43-years-old female with giant tumor-like LDH. (A, B, E) Plain MRI showing L_5_/S_1_ intervertebral disc sequestered protrusion with tumor-like changes. (C, D, F, G) On enhanced MRI, the sequestered nucleus pulposus can be seen with marginal annular enhancement and no enhancement centrally, suggesting a positive “bull’s eye” sign (Type A). (H, I, J) Plain MRI showing significant resorption of the protrusion. (K, L, M) Plain MRI showing complete resorption of the L_5_/S_1_ intervertebral disc protrusion, with a protrusion rate of 0% and a RR% of 100%. LDH = lumbar disc herniation, MRI = magnetic resonance imaging, RR% = resorption rate.

After an average follow-up of (23.00 ± 18.65) months, all patients had resorption, and the total RR% was (84.33 ± 12.57) %. Resorption occurs 1.5 to 12 months after treatment, with an average of (4.06 ± 3.32) months. The VP decreased from (1782.70 ± 1128.62) mm^3^ before treatment to (249.58 ± 224.04) mm^3^ at the last follow-up (*P* < .05). The AR% decreased from (47.20 ± 10.65) % before treatment to (10.70 ± 8.32) % at the last follow-up (*P* < .05), with an average improvement rate of (75.08 ± 22.29) %. Typical MRI images were shown in Figure 3

### 3.3. Outcome of clinical symptoms and follow-up

The clinical symptoms of all patients improved after non-surgical treatment. The JOA score increased from (12.88 ± 4.94) points before treatment to (26.25 ± 2.82) points after treatment (*P* < .05), with an improvement rate of (82.92 ± 15.43) %. During the treatment, no neurological damage occurred in all patients, and the clinical symptoms did not recur in the subsequent follow-up. Data for each patient was shown in Table 1.

**Table 1:** Patients’ characteristics.

Case no		1	2	3	4	5	6	7	8
Gender		F	F	M	M	M	M	F	M
Age (yr)		43	34	20	48	50	51	40	39
Course of disease		3 mo	8 mo	12 mo	3 wk	1 wk	1 mo	2 mo	1 mo
Protruding segment		L_5_/S_1_	L_4/5_	L_4/5_	L_4/5_	L_5_/S_1_	L_4/5_	L_4/5_	L_4/5_
Classification of “bull’s eye” sign		Type A	Type A	Type A	Type A	Type A	Type A	Type B	Type B
VP (mm^3^)	at the first visit	2518.55	1800.28	1638.74	4225.25	1000.52	1221.25	900.47	956.56
	at the last follow-up	0	684.11	250.73	422.53	0	256.46	166.59	216.18
	RR% (100%)	100	62.0	84.7	90	100	79	81.5	77.4
AR % (100%)	at the first visit	47.11	52.83	38.92	65.74	57.11	39.54	42.12	34.24
	at the last follow-up	5.12	8.62	27.2	8.03	4.29	3.29	19.24	9.78
	Improvement rate (100%)	89.13	83.68	30.11	87.79	92.49	91.68	54.32	71.44
JOA score (points)	at the first visit	16	7	19	4	13	14	15	15
	at the last follow-up	25	21	29	29	29	25	25	27
	Improvement rate (100%)	69.23	63.64	100	100	100	73.33	71.43	85.71
Time of resorption (mo)		3	3	2	1.5	3	4	12	4
Total follow-up time (mo)		29	3	44	52	30	4	14	8

AR% = herniated area as percentage of corresponding vertebral canal area, JOA = Japanese orthopaedic association, RR% = resorption rate, VP = volume of protrusion.

## 4. Discussion and conclusion

### 4.1. Clinical manifestations of LDH resorption

According to existing case reports, the time span for resorption is 2 months to 4 years after onset.^[4,10]^ Currently, it is generally considered that active resorption occurs within 6 months after onset. At this stage, patients’ clinical symptoms can be relieved, and some patients show obvious resorption or even disappearance of the herniated disc. Young and middle-aged patients have a high probability of resorption.^[11,12]^ Advanced age is not conducive to resorption owing to serious intervertebral disc degeneration, loss of water in the intervertebral disc tissue, and thickened annulus fibrosus.^[13]^

### 4.2. “Bull’s eye” sign in enhanced MRI

**Figure 4.**
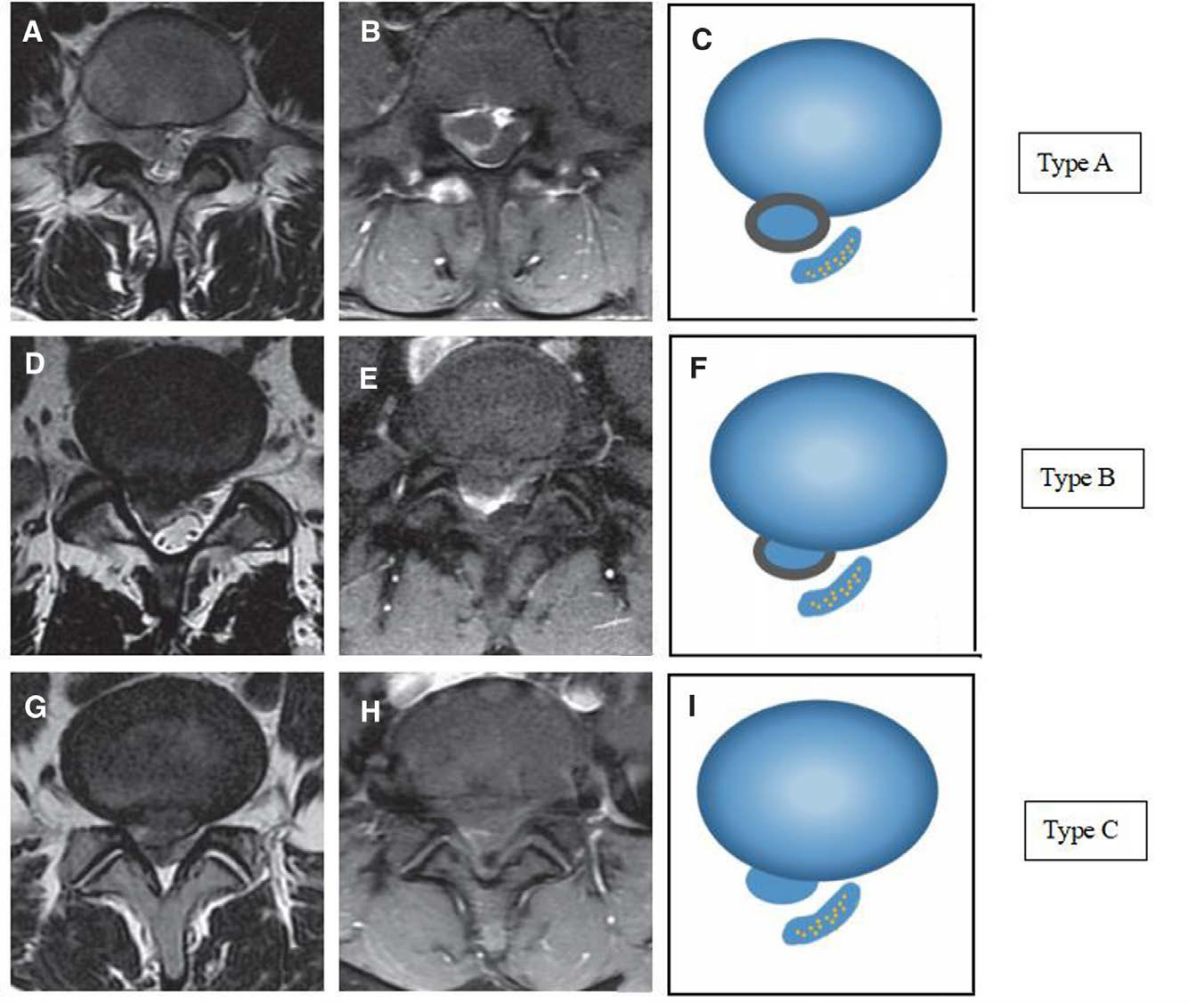
Classification of “bull’s eye” sign on enhanced MRI. (A, D, G) Maximum level of the plain MRI T2WI horizontal view; (B, E, H) Maximum level of the enhance MRI T1WI horizontal view; (C, F, I) Schematic diagram of the protrusion ring enhancement area: type A, showing a complete “bull’s eye” sign, the annular enhanced area surrounds the whole protruding nucleus pulposus; type B, showing an annular enhanced area partially surrounded the protruding nucleus pulposus; type C, there is no obvious annular enhancement around the protrusion. MRI = magnetic resonance imaging.

Through Our clinical observation, sometimes enhanced MRI shows the phenomenon of edge enhancement of protrusion (we call it “bull’s eye” sign) which was more prone to resorption. The growth of new blood vessels into herniated disc tissue (vascularization) and the formation of local inflammatory granulation tissue are the possible causes of “bull’s eye” sign.^[14]^ Because vascularization and inflammatory reaction are high signal in enhanced MRI, we believe that “bull’s eye” sign is the direct reflection of the pathological process of resorption in imaging, which can well predict the resorption probability. We selected the largest level of the herniated disc on the T1WI horizontal view of enhanced MRI, and divided it into 3 types according to the size of the marginal annular enhancement around the protrusion: type A, showing a complete “bull’s eye” sign, the annular enhanced area surrounds the whole herniated nucleus pulposus, and even the edge is thickened, showing a mass enhanced signal; In type B, the annular enhanced area partially surrounded the herniated nucleus pulposus, or only showed linear enhancement signal; In type C, there is no obvious annular enhancement around the herniated disc (Fig. 4). Clinically, there are also many reported cases of resorption with the “bull’s eye” sign after non-surgical treatment.^[15,16]^ Yu PF^[17]^ conducted clinical observation on 64 patients with LDH, including 26 patients with type A, 23 patients with type B and 15 patients with type C, they were followed up for 12 to 34 months. Finally, 42 patients completed non-surgical treatment. The success rates of non-surgical treatment of type A, type B and type C were 88.46%, 69.57% and 20.00%. Resorption occurred in 32 cases of type A and type B, while no resorption occurred in type C. In this report of 8 patients with resorption, the type of “bull’s eye” sign classification in all patients is type A or type B, which is consistent with the reports published by other scholars. This result increases our confidence in the success of the preferred non-surgical treatment for LDH patients with type A and type B.

### 4.3. Differentiation between intraspinal tumors and LDH

Intraspinal tumors tend to occur in young adults. Giant/sequestered LDH can cause cauda equina damage owing to stimulation of the dural sac or nerve root by the protruding tissue. The related clinical symptoms and signs are often atypical and easily confused with intraspinal tumors. Yu et al^[18]^ and Liu et al^[19]^ each reported 1 patient with low back pain. MRI showed an intraspinal tumor-like mass, and postoperative pathology showed intradural disc herniation. Enhance MRI is considered an effective imaging method to distinguish intraspinal tumors from LDH. Intraspinal tumors generally show that the signal intensity of the mass is inconsistent with intervertebral disc tissue, the boundary is clear, the mass is oval or dumbbell-shaped, internal density is uneven, and the condition may be accompanied by cystic degeneration. Giant/sequestered LDH generally shows that the signal intensity of the protruding tissue is consistent with that of intervertebral disc tissue, the boundary is irregular and unclear, and the intervertebral space at the responsible segment is narrowed. Giant/sequestered nucleus pulposus tissue can show protrusion marginal enhancement owing to vascularization of protruding tissue; that is, the “bull’s eye” sign. However, due to sufficient blood supply, tumor tissues usually show obvious uneven enhancement. Identifying them by different manifestations of enhancement is another reason for our non-surgical treatment of LDH based on enhance MRI.

### 4.4. Challenges of non-surgical treatment of giant LDH

To date, a number of cases of resorption in giant LDH have been reported.^[2,3]^ However, some scholars are worried about the risk of non-surgical treatment of giant LDH, such as cauda equina syndrome. According to our large sample of clinical observations, we found that, if patients were closely followed and observed with reference to the 3 conditions mentioned above, only 21.76% of patients with extruded and sequestrated LDH eventually received surgery.^[20]^ Therefore, through the combination of imaging examination and clinical symptoms and signs, we can analyze patients’ possible outcome trends. For patients with less serious symptoms and a high probability of resorption, non-surgical treatment can be attempted. Reviewing the historical process of the discovery and development of LDH, we believe that a clear understanding of the phenomenon of resorption in LDH and the promotion of this phenomenon through relevant interventions would have far-reaching implications for increasing the benefits to patients and society.

## Acknowledgments

The authors would like to appreciate all the staff of the participating scientific research.

## Author contributions

**Conceptualization:** Xueqiang Shen, Jintao Liu, Pengfei Yu.

**Data curation:** Shun Lin, Hong Jiang,Yu Zhu.

**Visualization:** Xueqiang Shen, Shun Lin, Yu Zhu.

**Project administration:** Xueqiang Shen, Jintao Liu.

**Resources:** Hong Jiang.

**Writing-original draft:** Xueqiang Shen.

**Writing-review & editing:** Jintao Liu, Pengfei Yu.
